# Stabilization and correction of aberrated laser beams via plasma channelling

**DOI:** 10.1038/s41598-024-62997-x

**Published:** 2024-05-27

**Authors:** Alexandre Rondepierre, Alexei Zhidkov, Driss Oumbarek Espinos, Tomonao Hosokai

**Affiliations:** 1 Institute of Scientific and Industrial Research (SANKEN), Osaka University, 8-1 Mihogaoka, Ibaraki, Osaka 565-0871 Japan; 2 Laser Accelerator R&D Team, Innovative Light Sources Division, RIKEN SPring-8 Center, 1-1-1, Kouto, Sayo-cho, Sayo-gun, Hyogo, Osaka 679-5148 Japan; 3 Present Address: Mitsubishi Electric Corporation, Advanced Technology R&D Center, Industrial Automation Systems Department, Laser Systems Section, Amagasaki, Japan

**Keywords:** Optical physics, Plasma physics

## Abstract

High-power laser applications, and especially laser wakefield acceleration, continue to draw attention through various research topics, and may bring many industrial applications based on compact accelerators, from ultrafast imaging to cancer therapy. However, one main step towards this is the arch issue of stability. Indeed, the interaction of a complex, aberrated laser beam with plasma involves a lot of physical phenomena and non-linear effects, such as self-focusing and filamentation. Different outcomes can be induced by small laser instabilities (i.e. laser wavefront), therefore harming any practical solution. One promising path to be explored is the use of a plasma channel to possibly guide and correct aberrated beams. Complex and costly experimental facilities are required to investigate such topics. However, one way to quickly and efficiently explore new solutions is numerical simulations, especially Particle-In-Cell (PIC) simulations if, and only if, one is confidently implementing such aberrated beams which, contrary to a Gaussian beam, do not have analytical solutions. In this research, we propose two new advancements: the correct implementation of aberrated laser beams inside a 3D PIC code, showing a great consistency, under vacuum, compared to the calculations with Fresnel theory); and the correction of their quality via the propagation inside a plasma channel. We demonstrate improvements in the beam pattern, becoming closer to a single plasma mode with less distortions, and thus suggesting a better stability for the targeted application. Through this confident calculation technique for distorted laser beams, we are now expecting to proceed with more accurate PIC simulations, closer to experimental conditions, and obtained results with plasma channels indicate promising future research.

## Introduction

In the recent years, there has been a tremendous blooming interest for high-power laser processes such as laser fusion, machining or laser-driven particle accelerators. For the latter one, the laser wakefield acceleration technique (LWFA, proposed by Tajima and Dawson in 1979^1^) is even considered as a potential candidate to provide X-ray free electron lasers^2,3^. However, for these highly non-linear processes, efforts are still required to go towards industrial systems and especially to improve both the stability and the quality of the generated electron beam. For instance, various schemes have been explored^4–6^, and, evidently, the driving laser part is a key component on which to play with^7^.

Nonetheless, real laser applications are often far from using simplified Gaussian beams, as disturbances can modify the wavefront, even if a lot of efforts and costs are often put on laser maintenance and beam quality control to keep the delivered laser as close as possible to the perfect diffraction limited case. However, it has been recently experimentally demonstrated that drastic changes arise when tuning the laser wavefront^8,9^, while other works previously started to explore this matter through a coupling between theory, simulations and experiments^10–13^. But, up to now and to the best of our knowledge, the numerical investigation of this arch-issue for LWFA remains limited with current PIC (particle-in-cell^14^) simulations. Other methods for laser filamentation have already been proven to work^15,16^, but they are not applicable to simulate common processes for LWFA. Though there have been former attempts in LWFA to simulate properly real laser beams^11–13,17^, e.g. including the wavefront distortion or focusing behaviour (retrieved from experimental measurements), yet it remains necessary to verify the conformity of the obtained results. As PIC simulations are utterly time consuming, the typical propagation distance of the process is limited to the millimetre scale thus preventing any use of the near-field laser input, usually well known and measured but not describable in an analytical way. Consequently, the true interaction of a high-power aberrated beam with a plasma can currently not be accurately described, and notably the issue of the halo phenomenon^10,18^ can not be addressed. In particular, Gaussian laser beams propagating inside a plasma channel can be converted into plasma waveguided modes, as explained under the paraxial approximation^19^, and we also believe that it may be true for aberrated beams. Hence, plasma channel may be a good candidate to mitigate the wavefront aberrations.

Therefore, it is essential to dispose of a validated method to propagate such beams and verify this kind of assumption. Various methods are possible to generate and shape a plasma channel^20,21^, making their practical application possible and thus their studies interesting. Former research demonstrated, using 2D PIC simulations, that a Gaussian beam can be well guided inside a parabolic plasma channel, and that distortions can be reduced compared to a non-parabolic plasma channel^22^, while the importance of the function that defines the plasma channel has been evidenced^23^.

In this article, we propose to use a method that enables us to confidently propagate aberrated laser beams inside a plasma channel, hence proving that even distorted beams can be converted into a single plasma mode. We confirm that such beams undergo notable improvements, especially regarding their intensity distribution. Since this technique is applicable to a broad range of distorted laser pulse (for which spatio-temporal effects are low as well as the wavelength dependence of the main laser characteristics) , we can extend this research further and evaluate the real parameter frame for optimizing such correction, with many possible applications in optics.

## Methods

The main approach followed in our work consists in properly implementing aberrated laser beam (transverse distribution) inside a 3D PIC simulation code. Indeed, arbitrary chosen distortions and laser patterns can give essential numerical effects if one can not guaranty their correct initialization and propagation.

Such PIC codes are suitable for laser matter interaction, however they are highly time consuming and only a few millimeters of propagation is possible in a reasonable amount of time with a supercomputer. Therefore, we have used the Fresnel diffraction integral to propagate a real laser beam from the near field region up to the vicinity of the focal region, after a few meters of propagation, where the PIC simulations starts. The fields calculated from Fresnel are then loaded and carefully set as initial conditions inside the PIC simulation, before starting the process of solving Maxwell’s equations.

**Figure 1:**
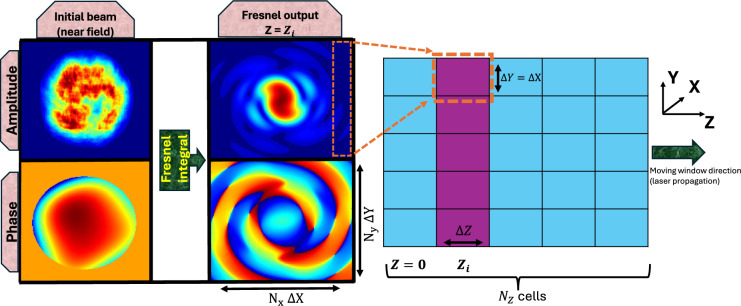
Procedure to set the initial field in a PIC code. Here, an experimental beam, chosen as the initial beam, has been propagated through Fresnel integral up to the distance that corresponds to the cell number $Z_i$, where the transverse field amplitude and phase is inserted. This method works with any kind of beam (either experimental and numerical-defined).

This choice has been made as experimental beams (both intensity and wavefront) are measured in the near field, and various set of aberrations can be added through Zernike polynomials formalism, as described in^10^. Furthermore, any theoretical beam shape (Gaussian, hyper-Gaussian, Bessel...) can also be implemented in the Fresnel propagator tool we developed and used. However, other classical methods could have been used such as the Rayleigh-Sommerfeld method or the angular spectrum method. The followed process has been summarized in Fig. 1.

### Fresnel propagator and aberrated beam description

Theoretically, any given phase map can be described using the decomposition with Zernike Polynomials, which is a family of polynomials that are orthogonal to each other on a unit disk. Thus the phase can be expressed as a linear combination of different polynomials^24–26^. For example, the 2nd and 3rd Zernike polynomials correspond to the tilt (0 and 45 degrees) while the 8th and 9th correspond to vertical and horizontal coma. However, it is worth noting that all these conventions are subjective and depend on the family of polynomials used to describe the phase map. Another family of polynomials is Seidel polynomials (that do not have the orthogonality property) and the polynomial used to describe astigmatism with this family will have a phase map slightly different from the one described with Zernike Polynomials^26,27^. Furthermore, Zernike polynomials are convenient when describing a circular beam (the orthogonality property is only verified on a unit disk), but for more complex shape (for instance, a square beam pattern) they will not be suitable.

The electric field E(x,y,$z_n$) of a laser beam propagating along the *z* axis at a given position $z_n$, if approximated as a monochromatic wave, may be written as:

1 $$\begin{aligned} E (x,y,z_n) = E_0 (x,y,z_n) \textrm{e}^{i(\omega t - k z_n)} \textrm{e}^{-i \varphi (x,y,z_n)} \end{aligned}$$

where $\omega$ is the wave angular frequency and k is the wave vector. $E_0$ is the amplitude and $\varphi$ is the phase spatially added to the beam, which includes both the aberration phase ($\varphi _a$) and the focusing phase ($\varphi _f$ = $\frac{k(x^2+y^2)}{2f}$), if needed.

In polar coordinates, the wavefront error phase is given by:

2 $$\begin{aligned} \varphi _a (\rho ,\theta , z_n) = \sum _{s=1}^{14} C_s (z_n) Z_s (\rho ,\theta ) \end{aligned}$$

where $C_s$ is a coefficient that represents the RMS amplitude of the aberration of index *s* over the whole beam, usually expressed in $\lambda$ units, and $Z_s$ is the s^th^ Zernike polynomial.

Thus, the total RMS value for the phase $\varphi _a$ $\sigma = \sqrt{\sum _{s=1}^{14} C_s^2 }$. More details can be found in^10^. This phase has to be calculated for any $z_n$ position to reconstruct a 3D real beam (slicing).

**Figure 2:**
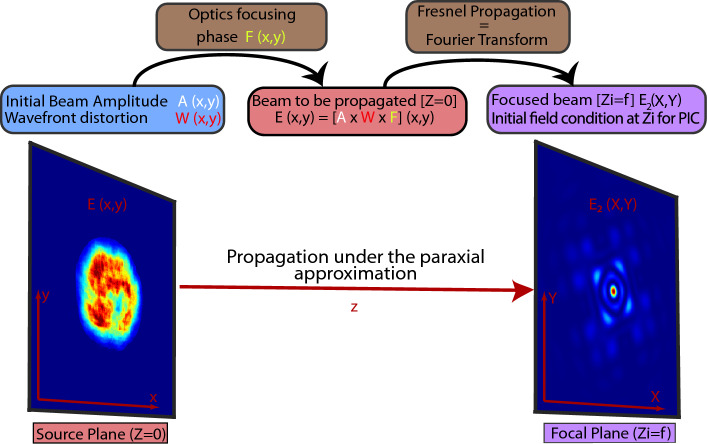
Global calculations conducted with the Fresnel propagator tool. The initial beam (amplitude and wavefront, left side) is propagated in a meters scale range before reaching the focal region, where multiple slices at $Z_i$ are extracted for their insertion inside the PIC code (right side).

Then, knowing the focal length *f*, the resulting far field beam $E_2$ in a given position $z_k$ (close to the focal plane in our case) is calculated through Fresnel integral:

3 $$\begin{aligned} E_2 (X,Y,z_k)&= \frac{\mathrm e^{i k z_k}}{i \lambda z_k} ~ \mathrm e^{i\frac{k}{2z_k}(X^2+Y^2)} ~ \iint _{-\infty }^{+\infty } \left[ E (x,y,0) ~ \mathrm e^{i\frac{k}{2z_k}(x^2+y^2)} \right] ~ \mathrm e^{-i\frac{2\pi }{\lambda z_k}(xX+yY)} \,dx \,dy \end{aligned}$$

where *E*(*x*, *y*, 0) is the initial beam, including both the amplitude and the phase. This calculation has been made using a home-made MATLAB code based on Fast Fourier Transform (FFT), as described in references^10,28^. This Fresnel propagator method is summarized (geometry and procedure) in Fig. 2.

Once the electric field amplitude, phase and polarization is known and defined, we retrieve the associated magnetic field using Maxwell’s equations (e.g: $\nabla \times E$ = $\partial _t B$).

In this work, the mainly used aberrations were spherical aberration (with the associated $Z_{4}^{0}$ Zernike polynomial), vertical astigmatism ($Z_{2}^{-2}$), horizontal coma ($Z_{3}^{1}$) and trefoil 0^∘^ and 30^∘^ ($Z_{3}^{-3}$ and $Z_{3}^{3}$) and aberration were added to an initial Gaussian intensity distribution. The total wavefront distortion value (in RMS wavelength values) was set from $\lambda$/10 to $\lambda$/5.

It is worth noting that the Fresnel propagator tool gives the input for the PIC code at one unique wavelength. If one is interested in making a complete description, the Fresnel propagator tool can be run for multiple wavelengths from which the calculated input could be weighted according to the considered laser spectrum. However, to spare time and as this point is not critical, we took only a single wavelength. Especially the phase and the intensity distribution for all the considered wavelengths (with a typical bandwidth of about 50 nm), should not differ so much as the laser was amplified and sent to the target through the same optical path, and focused with achromatic optics.)

### 3D PIC simulation and implementation of aberrated beams

The 3D relativistic PIC code FPLaser^29–31^ was used to perform the simulations. Each simulation uses a moving window (moving at the speed of light) of 120 $\times$ 120 $\upmu$m in transverse size, and 25 $\upmu$m in longitudinal size (direction of propagation), and is based on the resolution of Maxwell’s equations. The number of cells used in the following simulations are 756 in each transverse direction and 500 in the longitudinal one, with 8 particles per cell. The spatial step between two iterations is 50 nm. Calculations of about 3 mm of propagation are conducted using a supercomputer with 2916 cores taking about 80 h.

In PIC codes, one has to define the initial laser beam used for the simulation. The standard way is to use a beam with an existing analytical solution, e.g. a Gaussian one using the parabolic equation and paraxial approximation^32,33^. However, for a distorted beam, such equations are not available and one has to properly define the real beam so that it propagates inside the plasma with confidence. To reach this objective, we use the previously calculated transverse field outputs (both amplitude and phase, produced from Fresnel calculation as shown in Fig. 2), and we load them (as an initial conditions for the fields) inside the PIC simulation as follows:

- First of all, using the previous Fresnel propagator (Eq. 3), one has to define the initial intensity and wavefront error distribution in the near field (for example, using Zernike formalism). Either an experimental case (for instance measured with a camera and a wavefront sensor^8^, or numerically reconstructed^11,12,34^) or a theoretical case (for instance a top-hat beam with a wavefront error of $\lambda$/10 for astigmatism) can be used.
- Then, for implementing and reproducing the real beam used in our simulation, it is required to extract at least $N_Z$ = 500 slices (with a resolution of $\{N_X \times N_Y \}=\{756\times 756\}$ for the transverse field) from Fresnel, corresponding to the $N_Z$ different positions of the PIC windows, separated by 50 nm. This slicing is compulsory to ensure a correct initialisation of the beam before starting the PIC simulation. These slices are then integrated inside the PIC code as initial conditions for the field equations before solving Maxwell’s equations.

As summarized in Fig. 1, it is worth noting that the same resolution obtained after Fresnel calculation (either for the spacing slice to slice or for the transverse field) has to be used inside the PIC code.

The laser wavelength is unique and set to $\lambda$ = 800 nm (frequency $\omega$ in Eq. 1), and an exponential temporal envelope (FWHM = 25 fs, which corresponds to 7.5 $\upmu$m spatially) is used to define the beam duration in the simulation. The laser is set linearly polarized along either the X or Y direction.

The main parameters used for the simulations are summarized in the Table 1.

**Table 1:** Main parameters used in the PIC simulation.

Wavelength (nm)	Pulse duration (fs)	Intensity (GW/cm^2^)	X/Y size ($\upmu$m)	Z length ($\upmu$m)	Particles per cell
800	25	8 $\times$ 10^18^	120	25	8

### Plasma channel inside 3D PIC code

**Figure 3:**
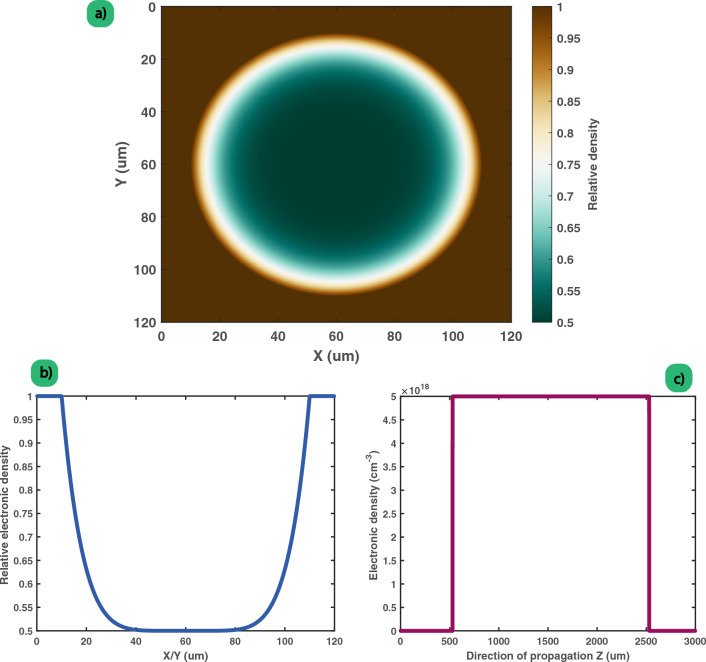
Example of a 2 mm length plasma channel used in the PIC simulation with sextic (R^6^) function for the gradient. (**a**) Transverse relative electronic density ((X,Y) plane); (**b**) 1D profile along either X or Y direction, taken at the center of the channel; (**c**) Evolution of the electronic density as function of the propagation distance Z in the case of a maximum density of 5 $\times$ 10^18^ cm^-3^.

As shown in Fig. 3, the used plasma channels were designed as a straight path (along the direction of propagation of the beam, Z) with a circular (thus symmetric) density drop in the (X,Y) plane of 100 $\upmu$m of diameter, centered inside the simulation window. The circular aperture has a maximum depth of half the outside density $n_o$ (taken from $10^{18}$ to $10^{19}$ cm^-3^) , and the radial profile chosen is polynomial ($R^N$, where $R^2$ = $X^2+Y^2$ and N chosen from 2 to 6).

Optically, as the refractive index seen by the laser is evolving opposite to the electron density ($n = \sqrt{\frac{n_c-n_e}{n_c}}$, with $n_c$ the critical density), this plasma channel corresponds to a graded index fiber.

The density along the direction of propagation evolves linearly (up-ramp and down-ramp of 30 $\upmu$m, with a flat length region in between of about 2 mm) from 0 to $n_o$, and starts after 500 $\upmu$m of propagation (thus, 500 $\upmu$m before the laser focal position in vacuum). The self-focusing threshold power^35^ is approximately 15 TW for the presented simulations, while the laser power is set around 25 TW. The Rayleigh length of the used laser beam is about a fifth of the plasma length, around 400 $\upmu$m.

Such plasma channels may be generated with a pre-pulse laser or a discharge inside a gas, but these mechanisms are not the topic of our research.

## Results

### Confident propagation of aberrated beams

#### Comparison between PIC and Fresnel

To ensure that our PIC code is capable of propagating the inserted aberrated beam, we checked, under vacuum, that the beam pattern and shape after a few millimeters of propagation were consistent with the output generated by the Fresnel propagator at these positions as well.

**Figure 4:**
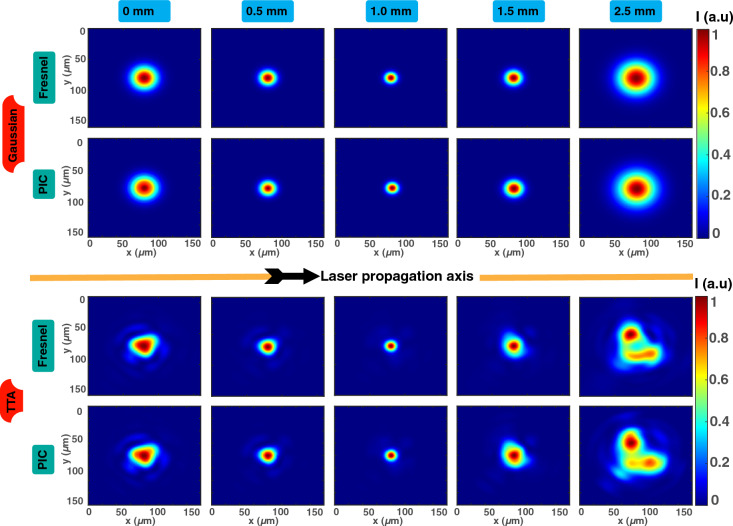
Comparison of the results obtained from Fresnel calculation and with conventional PIC simulation in the case of a Gaussian beam without aberrations (top side) and in the case of an aberrated beam (bottom side, TTA configuration (astigmatism and trefoil, as described in^8^)). From left to right, the laser is propagated and the normalized transverse intensity ((X,Y) plane) is plotted up to 2.5 mm of propagation. A high consistency between the two methods is obtained.

In Fig. 4, we have plotted the normalized transverse intensity of the laser beam for 5 different positions (0 mm, 0.5 mm, 1 mm, 1.5 mm and 2.5 mm). The 0 mm position, which is the beginning of the PIC simulation, is located 1 mm before the focal position. Intensities patterns are compared between the Fresnel’s theory and the PIC simulation, and great consistency is shown either with the Gaussian case (no aberration) or with the TTA configuration (experimentally used in reference^8^). This latter configuration mixes trefoils (0 and 30) and astigmatism and its complexity helps to ensure the proper behaviour of our simulations run on the FPLaser 3D PIC code. It confirms that distorted beams can be correctly propagated with the PIC code. However, it is worth noticing that either Fresnel diffraction or PIC code is numerically performed with a limited resolution, so that we can expect some very small differences between the two methods, as observed for the TTA case. Fresnel calculations are also conducted under the paraxial approximation at a single wavelength. Altogether, the outputs produced with the Fresnel propagator, which is a very established and verified method, are consistent with the ones produced with the PIC simulation in which the initial conditions, a few millimeters before the position of comparison, where initialized with the beam produced from Fresnel propagator and propagated with the resolution of Maxwell’s equations. Therefore, it confirms that this method is suitable to propagate various aberrated beam transverse distribution with the correct phase behavior inside a PIC code. To the best of our knowledge, this is the first time that such implementation has been conducted and verified with a robust method.

#### Comparison between aberrated beams and the Gaussian beam

**Figure 5:**
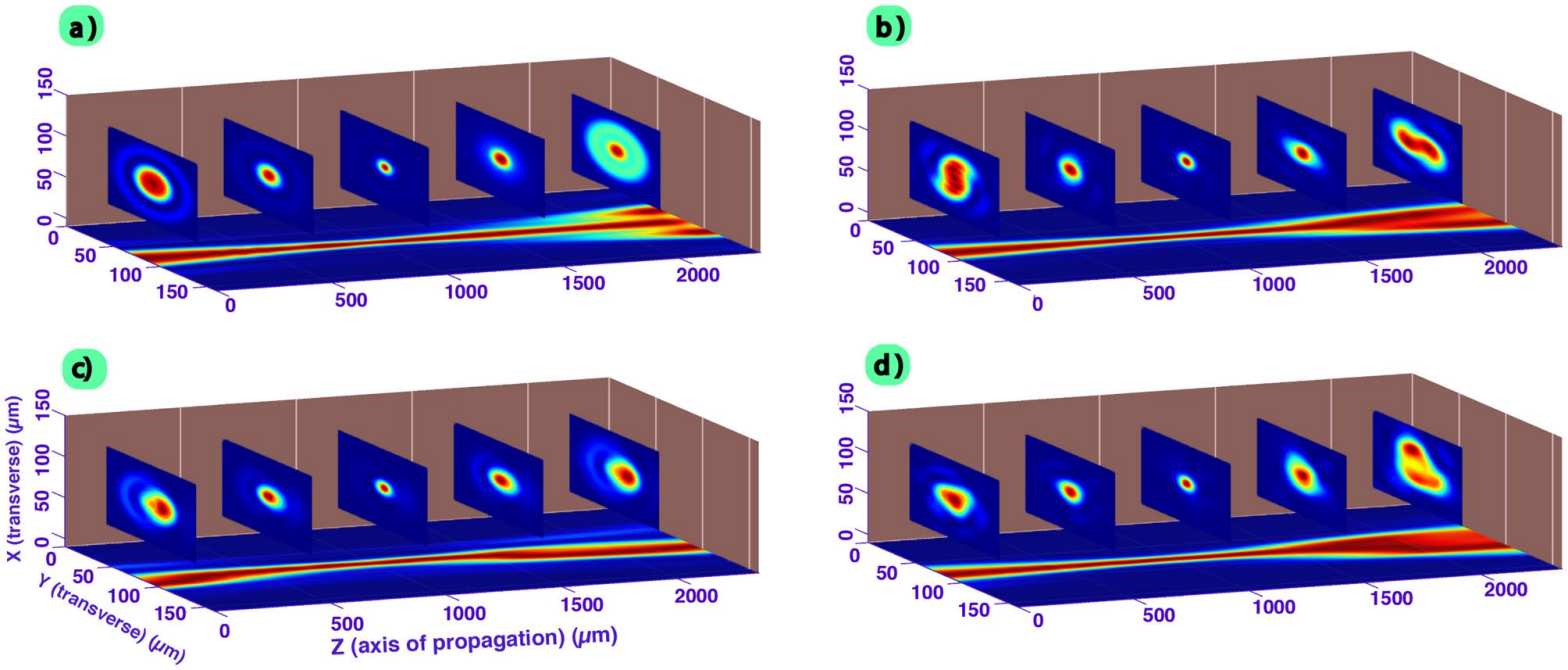
Normalized transverse intensity profiles of the laser ((X,Y) plane) for 5 different positions along the direction of propagation (0, 0.5, 1, 1.5 and 2 mm). The normalized 2D intensity profile ((Y,Z), taken at X/2) as function of the propagation distance is projected below. In each cases (**a**) Spherical aberration, (**b**) astigmatism 0, (**c**) horizontal Coma, (**d**) TTA a total RMS value of $\lambda$/10 for the wavefront error is used.

As already mentioned in the introduction, being able to properly propagate an aberrated (thus, realistic) laser beam, including the focusing behaviour, could be a great asset for simulations of high-power laser processes. Particularly, this is often desired for PIC simulations and consequently we have plotted in Fig. 5 a set of results obtained for different kind of aberrations (spherical one, coma, astigmatism and the TTA). The normalized transverse intensity profile is plotted as a function of the propagation distance and the 1D-cut intensity profile (taken in the middle of the window, at X/2) during the propagation is projected on the (Y,Z) plane.

It is worth noting that the behaviour of the various beams (pattern evolution during propagation) is absolutely different depending on the considered aberration, as expected from recent experimental results^8^. Altogether, it confirms that there is a clear need to expand such research, as running PIC simulations for LWFA with such beams may provide important results and understanding. It should be also possible to find great explanations on which configurations can improve LWFA, for instance.

### Improvement of aberrated beams via plasma channeling

We have proposed to investigate if a real laser beam can be corrected when propagating inside a plasma channel. Since we demonstrated that aberrated laser beams are correctly implemented inside our 3D PIC simulation code, thus we can use this technique to confirm whether and how far a distorted beam can be guided and if some alterations or improvements results from this propagation, as it may be converted into a single plasma mode.

#### Improvements of aberrated beams: single mode plasma conversion

A typical aberrated beam, represented by the previously mentioned TTA configuration, was propagated in vacuum and in various plasma channels.

**Figure 6:**
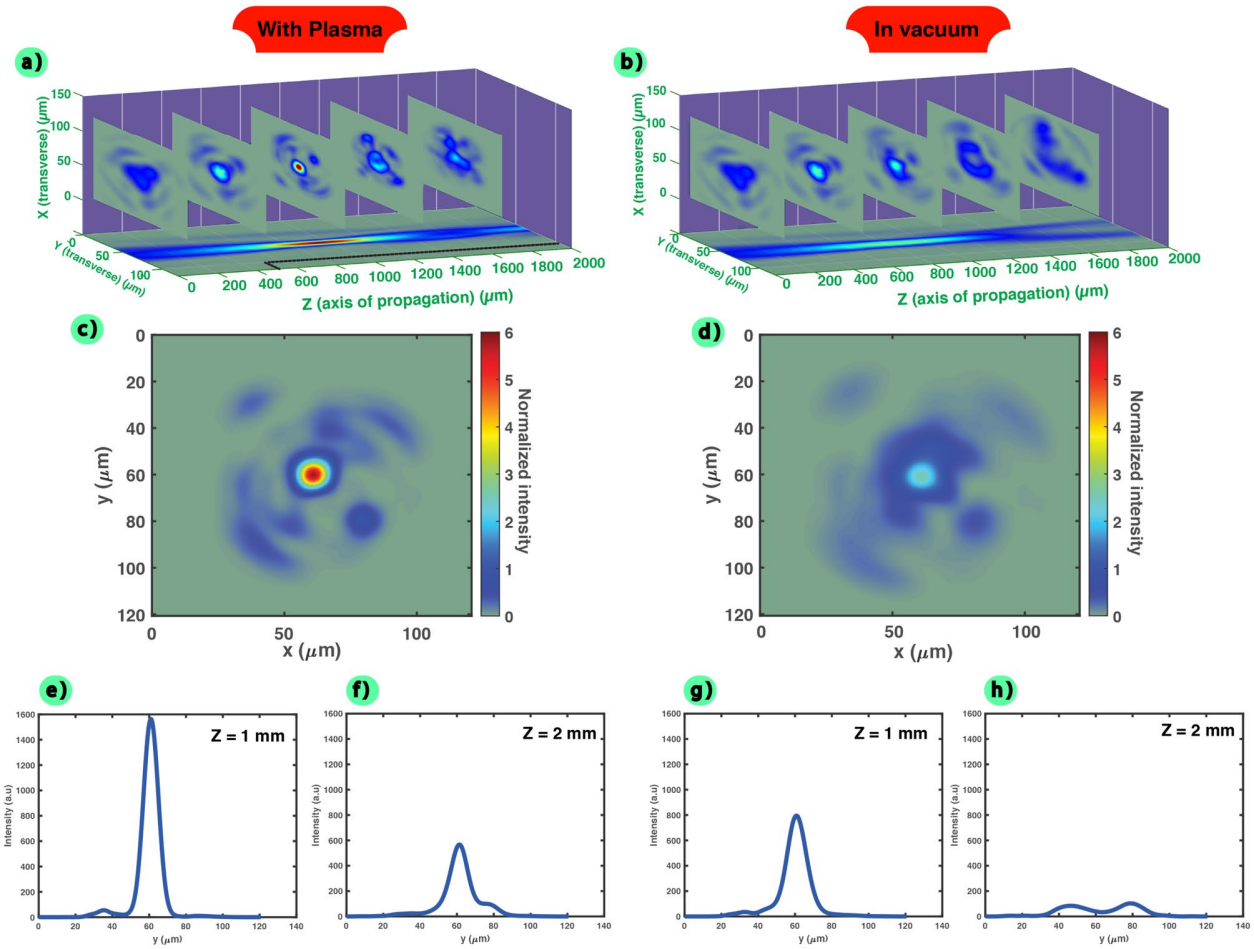
Comparison of the aberrated laser propagation (TTA configuration, total RMS wavefront error of $\lambda$/5) in vacuum or in plasma in the case of a sextic channel profile ($R^6$) and with a density of 5 $\times$ 10^18^ cm^-3^. (**a**) and (**b**) corresponds to the normalized transverse intensity profiles of the laser ((X,Y) plane) for 5 different positions along the direction of propagation (0, 0.5, 1, 1.5 and 2 mm). The normalized 1D intensity profile ((Y,Z), taken at X/2) as function of the propagation distance is projected below (the normalization has been conducted using the maximum value reached in the case of the propagation inside the plasma and the plasma position is shown in black dashed lines.). The transverse intensity profile obtained at the waist position are shown in (**c**) for plasma and in (**d**) for vacuum. The 1D intensity profile after 1 mm of propagation (**e**) and 2 mm (**f**) inside the plasma are also plotted (respectively (**g**) and (**h**) in vacuum).

As shown in Fig. 6, the total RMS error for the wavefront is set to $\lambda$/5 and a sextic plasma channel (half-density drop evolving as $R^6$ from 5 $\times$ 10^18^ cm^-3^ ) is used. In that case, it can evidently be observed that the behaviour between the vacuum propagation and the plasma one is highly different (a) and (b), in particular the beam is well guided and corrected when going through the plasma channel. Moreover, when observing the intensity distribution at the waist position, the aberrated beam is showing a better pattern, closer to a Gaussian pulse, with a central maximum intensity higher than the one measured under vacuum. The beam seems to be converted into a single plasma mode, even though its pattern can not be perfectly corrected in that case, especially when propagating after the focal region. This is also confirmed by the 1D transverse intensity profiles at the waist position and 1 mm after (e) and (f) for the plasma channel; (g) and (h) under vacuum. The beam inside the plasma channel is showing a distribution close to a Gaussian one, compared to the propagation under vacuum. At the waist position, the maximum reached intensity inside the channel is increased by 2 times compared to the propagation under vacuum. After 1 mm of propagation from the waist, this value is about 6 times. Thus, this shows that the beam and its halo are better focused.

**Figure 7:**
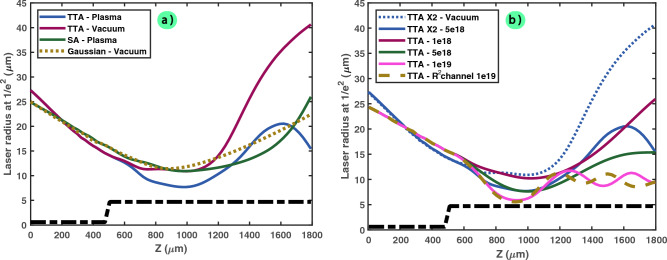
Laser radius size (at (1/e)^2^ intensity, in $\upmu$m) as function of the distance of propagation ($\upmu$m). (**a**) The TTA and Spherical Aberration (SA) configurations are compared with the Gaussian case (aberration free) in vacuum and in plasma. (**b**) The effect of various plasma densities (From 10^18^ to 10^19^ cm^-3^), plasma shapes (one case with $R^2$, else $R^6$) or amplitude of aberration (total RMS value of $\lambda$/10 or $\lambda$/5) on the TTA configuration is shown. The plasma position is plotted in black dashed lines.

It is worth noting that such behaviours were also observed when implementing other distributions of aberration (Fig. 7a), thus indicating that such plasma channels could convert (and then, stabilize) the incoming aberrated beams into a single plasma mode (Gaussian-like profile, with a better energy concentration). Indeed, the case presented in Fig. 6 has been taken as a sample of a typical random set of aberrations, however the beam pattern at the waist (c) for the 2D distribution and e) for the 1D profile) is converging to the same structure for the multiple aberrations we tried (spherical, astigmatism, or others) and for any range of amplitude (from $\lambda$/5 to $\lambda$/10).

#### Effect of the plasma channel properties

In Fig. 7b, the effect of the maximum plasma density (chosen from 10^18^ to 10^19^ cm^-3^) and of the plasma channel function (either $R^2$ or $R^6$ function) is investigated by calculating the laser waist size as function of the propagation distance.

When increasing the density, thus reinforcing the self-focusing effect notably, it can be observed that the laser size becomes smaller. On the other hand, there is no noticeable effects of the plasma channel function on the laser size. However, some small oscillations start to appear when changing the density, and their frequencies depend on the plasma function, which is consistent with previous calculations^36^ and simulations of parametric resonance^37^.

In all cases, it is demonstrated that the plasma channel is able to concentrate the energy by reducing the laser size compared to the propagation inside vacuum.

## Discussion

Recent experimental results demonstrated the importance of the wavefront error for laser wakefield acceleration, even with some improvements obtained, which was counter-intuitive. As this influence of laser aberrations could also extend to multiple high-power applications, we have presented here a method to simulate the propagation of a typical aberrated laser beam inside plasma by using a 3D PIC code based on Maxwell’s equations solving. The Fresnel integral method was used to conveniently propagate a near field, well-known beam to the vicinity of the focal region, where the PIC simulation starts, before extracting the initial field conditions. Still using Fresnel’s theory, such inserted aberrated beams were propagated through the PIC simulation and they show a great consistency with the proven Fresnel method.

Such confidence in propagating real distorted beams has paved the way for the correct study of the propagation of these lasers inside plasma channels. Indeed, we have proposed and assumed that these kind of parabolic plasma channels should be able to improve even aberrated beams by converting them into a single plasma mode. Our presented numerical method has allowed us to draw clear and correct conclusions.

We have underlined that the propagation behaviour is drastically modified when going through such plasma channels. In this instance, improvements of various aberrated beams were observed and seems related to a conversion into a single plasma mode. The intensity concentrated in the main spot was increased by up to 2 times in the waist position, and up to 6 times 1 mm after the waist. The halo disturbance around the main central Gaussian spot has also been reduced.

Though a perfect correction could not be obtained, which was far from the goal of this work (we have explored the channel’s capability of correction rather than optimizing its parameters), one may consider this plasma channelling as a way to improve the quality of distorted lasers. Indeed, this work aimed at demonstrating that an arbitrary chosen plasma channel (although carefully initialized) can improve incident aberrated beams by producing the same laser output in a given region. Consequently, unstable (e.g wavefront) incoming laser pulses would give the same electron beam parameters. Altogether, as shown by the presented results when implementing the plasma channel, the objective was bring stability rather that perfectness of the laser pulses in their focus point.

Therefore, this conversion into a single mode plasma could also be a solution to enhance the stability of many applications, as the fluctuations of the wavefront could be thwarted: any incident laser pulse should convert to the typical pattern obtained in Fig. 6c,e.

Altogether, future works should investigate this method with a broader range of parameters (plasma length, position and properties, mainly). In particular, some improvements can still be conducted from the laser side by considering a real longitudinal distribution (and not a perfect Fourier-transformed-limited pulse) and eventually modeling the phase and intensity distribution for different wavelengths. Furthermore, it could also be interesting to investigate spatio-temporal coupling issues such as angular chirp even if it was experimentally demonstrated that conventional values (i.e when the laser compressor system is correctly aligned) are not enough to play a role in process such as LWFA or laser proton acceleration^38,39^. This would help to find an optimal configuration that should stabilize any incident aberrated laser into this single mode plasma. Such plasma channels might be a key component for the development of plasma optics.

## Data Availability

Data underlying the results in this paper are not publicly available but may be obtained from the authors upon request.
